# Bioinformatics Analysis Identified miR-584-5p and Key miRNA-mRNA Networks Involved in the Osteogenic Differentiation of Human Periodontal Ligament Stem Cells

**DOI:** 10.3389/fgene.2021.750827

**Published:** 2021-09-27

**Authors:** Chengze Wang, Lingling Dong, Ying Wang, Zhiwei Jiang, Jing Zhang, Guoli Yang

**Affiliations:** Key Laboratory of Oral Biomedical Research of Zhejiang Province, The Affiliated Hospital of Stomatology, School of Stomatology, Zhejiang University School of Medicine, Hangzhou, China

**Keywords:** periodontal ligament stem cells, osteogenic differentiation, bioinformatics analysis, miRNA-mRNA networks, miR-584-5p

## Abstract

Human periodontal ligament cells (PDLCs) play an important role in periodontal tissue stabilization and function. In the process of osteogenic differentiation of PDLSCs, the regulation of molecular signal pathways are complicated. In this study, the sequencing results of three datasets on GEO were used to comprehensively analyze the miRNA-mRNA network during the osteogenic differentiation of PDLSCs. Using the GSE99958 and GSE159507, a total of 114 common differentially expressed genes (DEGs) were identified, including 62 up-regulated genes and 52 down-regulated genes. GO enrichment analysis was performed. The up-regulated 10 hub genes and down-regulated 10 hub genes were screened out by protein-protein interaction network (PPI) analysis and STRING in Cytoscape. Similarly, differentially expressed miRNAs (DEMs) were selected by limma package from GSE159508. Then, using the miRwalk website, we further selected 11 miRNAs from 16 DEMs that may have a negative regulatory relationship with hub genes. *In vitro* RT-PCR verification revealed that nine DEMs and 18 hub genes showed the same trend as the RNA-seq results during the osteogenic differentiation of PDLSCs. Finally, using miR-584-5p inhibitor and mimics, it was found that miR-584-5p negatively regulates the osteogenic differentiation of PDLSCs *in vitro*. In summary, the present results found several potential osteogenic-related genes and identified candidate miRNA-mRNA networks for the further study of osteogenic differentiation of PDLSCs.

## Introduction

The high incidence of periodontal disease is an important cause of tooth loss. Periodontal disease can cause varying degrees of periodontal bone defects. To date, various conventional therapies for periodontal regeneration have shown limited clinical outcomes. In clinical work, the main goal of periodontal surgery is to remove infections and restore defective tissues. Periodontal regenerative therapy with membranes and bone grafting materials called guided bone regeneration (GBR) and guided tissue regeneration (GTR) has been employed with distinct levels of clinical success. Surgery can help remove the source of infections but it is often difficult to recover periodontal ligament and alveolar bone. Literature studies (Kämmerer et al., 2017; Hu et al., 2018; Liu et al., 2019b) have reported that injection therapy based on PDLSCs promoted the regeneration of alveolar bone and periodontal ligament. Repairing alveolar bone defects and periodontal ligament requires the participation of PDLSCs. Understanding the signal pathway regulation in the process of osteogenic differentiation of PDLSCs can provide a theoretical basis for periodontal tissue regeneration.

Human PDLSCs were first isolated and reported by Seo et al. (2004). Recent studies have shown that PDLSCs may offer a more reliable strategy for the treatment of periodontal defects by a cell-based tissue engineering approach (Ma et al., 2017; Zhang et al., 2019; Aprilianti et al., 2020). This treatment method relies on the multiple functions of PDLSCs such as anti-inflammatory properties (Nagata et al., 2017; Liu et al., 2019a; Qiu et al., 2020), osteogenic differentiation ability, and chemokines secretion capacity (Lee J. S. et al., 2017; Wang et al., 2018). The osteogenic differentiation of PDLSCs is the most critical among them. Many studies have shown that PDLSCs also have an impressive effect on the regeneration of bone tissue. For example, PDLSCs are used for alveolar bone regeneration (Lee J. et al., 2017), which can restore bone defect by mixing hydroxyapatite (Aprilianti et al., 2020), tricalcium phosphate (Xu X. Y. et al., 2019) and hydrogels (Ma et al., 2017). In addition, the PDLSCs membrane sheet technology can promote the osseointegration of alveolar bone and implant (Iwasaki et al., 2019). In particular, one study (Zhang et al., 2018) has shown that human PDLSCs have similar osteogenic differentiation ability compared with bone marrow-derived MSCs (BMSCs) and adipose-derived stem cells (ADSCs).

In the past, it was reported that the osteogenic differentiation of PDLSCs was mainly related to the Wnt (Gu et al., 2017), integrin (Tang et al., 2017), and PI3K-AKT (Xu X. et al., 2019) signaling pathways. However, the specific molecular regulation mechanism of osteogenic differentiation of PDLSCs is still not clear. Recently, it has been reported that the miRNA-mRNA network also plays an important regulatory role in the osteogenic differentiation process of PDLSCs (Whitfield et al., 2004; Ahmad et al., 2021). Therefore, the analysis of multiple RNA-seq and miRNA-seq datasets is conducive to detailed screening of the changes in signal pathways during the osteogenic differentiation of PDLSCs.

Microarray techniques and bioinformatics analysis have been widely used to screen for the DEGs, functional pathways and PPI associated with osteogenic differentiation of stem cells (Yang et al., 2019; Fan et al., 2020; Bini et al., 2021; Shin et al., 2021). In this study, three microarray datasets (total 16 samples) were downloaded from Gene Expression Omnibus (GEO) for analysis to identify DEGs and DEMs between the control group and the induction group. In summary, 18 hub genes and nine miRNAs were verified consistently by qPCR *in vitro*. Among the nine miRNAs, we predict that miR584-5p can bind to ALPL and may regulate the expression of ALPL. ALPL is a very important osteogenic regulatory gene. Liu reported (Liu et al., 2020) that miR-584-5p was also down-regulated during osteogenic differentiation of dental pulp stem cells, which was in line with our sequencing analysis results. However, the article did not do further functional verification experiments of miR-584-5p. So we further verified the role of miR-584-5p in PDLSCs Osteogenic differentiation.

## Materials and Methods

### Microarray Data

Gene Expression Omnibus^1^ is a public functional genomics data repository. Before the study, we searched for related datasets on GEO’s website. On GEO’s website, we conducted a search using keywords “periodontal ligament stem cell” or “PDLSC” or “dental stem cell” or “periodontal ligament tissue” and “osteogenic differentiation.” We selected datasets including human PDLSCs samples with osteogenic differentiation and samples under normal medium as control. We selected GSE99958, GSE159507, and GSE159508 for further analysis. This study used three datasets, two mRNA-seq (GSE99958, GSE159507) and one miRNA-seq (GSE159508). GSE99958, GSE159507, GSE159508 were downloaded from GEO. GSE99958 contains four mRNA-seq samples, which are cultured in control medium GSM2666465, in osteogenic medium for 3 days GSM2666463, 7 days GSM2666464, and 14 days GSM2666462. GSE159507 contains 6 samples, Group Control (GSM4831419, GSM4831420, and GSM4831421) are cultured in control medium, Group Induced (GSM4831416, GSM4831417, and GSM4831418) are cultured in osteogenic medium for 14 days. GSE159508 contains six samples, three samples are under osteogenic induction for 0 days (GSM4831425, GSM4831426, and GSM4831427), and three samples are under osteogenic induction for 14 days (GSM4831422, GSM4831423, and GSM4831424). Database GSE99958 was performed on GPL17303, database GSE159507 was performed on GPL16956 Agilent-045997 Arraystar human lncRNA microarray V3 (Probe Name Version) and database GSE159508 was performed on miRCURY LNA microRNA Array, 7th generation; lot 35,106–35,106 (miRBase 21.0). Based on the platform annotation information, probes were transformed into corresponding gene symbols in the R software environment.

### Differentially Expressed Analysis

For GSE99958, GSE159507, GSE159508, the probe sets without corresponding gene symbols or the genes with multiple probe sets were removed or averaged, respectively. For the dataset GSE99958, without biological replicates, CORNAS (default 1.5-fold count change) was used to obtain DEGs (Hu et al., 2018). The DEGs of 3, 7, and 14 days compared to 0 days were obtained and the common DEGs were determined. For the dataset GSE159507, the limma package was used to identify DEGs. LogFC > 1 or LogFC < −1 and *P*-value < 0.05 were considered to indicate statistical significance. At last, the common up-regulated genes and down-regulated genes between GSE99958 and GSE159507 were selected. For the dataset GSE159508, the limma package was also used to identify the differentially expressed miRNAs (DEMs). Up-regulated miRNA (LogFC > 1 and *P*-value < 0.05) and down-regulated miRNA (LogFC < −1 and *P*-value < 0.05) were selected.

### Gene Ontology and KEGG Enrichment Analyses of Differentially Expressed Genes

We performed gene ontology (GO) analysis of common DEGs (62 up-regulated genes and 52 down-regulated genes) between GSE99958 and GSE159507. GO and KEGG enrichment analyses were performed *via* the package clusterProfiler on R platform. GO enrichment analysis predicted the functional roles of DEGs based on three aspects, including biological processes (BP), cellular components (CC), and molecular functions (MF). The threshold was *P* < 0.05.

### Protein-Protein Interaction Network Network Analysis and Analysis of Hub Genes

The protein-protein pairs of DEGs (62 up-regulated genes and 52 down-regulated genes) were identified *via* STRING (Szklarczyk et al., 2015) v11.0^2^. Then, the PPI networks were visualized in Cytoscape version 3.8.2 software^3^. CytoHubba, a plugin in Cytoscape, is used to screen hub genes, and the Degree algorithm is to select genes with the top 10 nodes ranked by degree.

### Targets Prediction for Differentially Expressed miRNAs and Candidate miRNA Selection

Potential targets for DEMs were predicted by bioinformatics algorithms in the miRWalk database (Sticht et al., 2018): miRWalk^4^. Because there are many target genes from miRNA prediction and the results of the algorithm prediction have large false positives. A negative regulatory relationship between hub genes and miRNAs is used to narrow the size of candidate miRNAs from DEMs.

### Isolation and Characteristics of Human Periodontal Ligament Stem Cells

The study protocol was approved by the Medical Ethical Committee of School of Stomatology, Zhejiang University and written informed consent was obtained from each individual patient before teeth extraction. Three healthy human premolars, which were extracted for orthodontic reasons, were used for periodontal ligament (PDL) cell isolation. Human PDL tissue was scraped from the middle third of the root surface and isolated as described by our previous article (Wang et al., 2017, 2020). Briefly, Each PDL tissue sample was cut into 1–2 mm fragments, and each fragment was placed in a T25 culture flask containing a minimum amount of α-MEM medium and 10% fetal bovine serum. The medium was changed every three days until the growth of PDLSC was observed. The cells (Supplementary Figure 1) were detached with trypsin-EDTA (0.25%) and cultured into fresh plates.

For adipogenic differentiation assays (Cyagen, China), cells were exposed to an adipogenic medium; the medium was a-MEM containing 10% FBS, 2 mM dexamethasone, 0.2 mM indomethacin, 0.01 g/L insulin, and 0.5 mM isobutyl-methylxanthine. The adipogenic medium was refreshed every 3 days. After 4 weeks, cells were stained with oil red O (Supplementary Figure 1E).

For chondrocyte differentiation (Cyagen, China), the medium contained a-MEM supplemented with 10% FBS, 2 ng/mL transforming growth factor-b1, ITS + Premix, 50 mg/mL L-ascorbic acid, 100 mg/mL sodium pyruvate, 100 nM dexamethasone, and 100 U/mL penicillin/streptomycin. After 4 weeks, chondrogenic differentiation was assessed *via* Alcian Blue (Solarbio) staining (Supplementary Figure 1C).

The inhibitor NC, miR584-5p inhibitor, mimics NC and miR584-5p mimics were synthesized by Sangon (Shanghai, China). PDLSCs were seeded at a density of 5 × 10^4^ cells/ml. The inhibitor NC, miR584-5p inhibitor, mimics NC and miR584-5p mimics were transfected into cells using jetPRIME according to manufacturer’s instructions (Invitrogen, United States). Total 50 nM artificial miRNA, 50 μL buffer and 0.5 μL jetPRIME per well were used in a 12-well plate. The medium was replaced after 24 h transfection.

### Alizarin Red and ALP Staining Assay

Periodontal ligament stem cells were plated into 12-well plates at 2 × 10^5^ cells per well for ALP staining assays and at 1.5 × 10^5^ cells per well for Alizarin Red staining assays. Cells were cultured in the osteogenic medium, which contains Alpha Modification Minimum Essential Medium Eagle (α-MEM) containing 10% Fetal Bovine Serum (FBS, GEMINI Bio, Liverpool, United Kingdom), 50 μM ascorbic acid, 10 μM Dexamethasone and 10 mM β-glycerophosphate. For ALP staining assays, PDLSCs were stained at 3 and 7 days with an alkaline phosphatase kit (Beyotime, China). For Alizarin Red staining assays, PDLSCs were stained with 2% alizarin red S (ScienCell, United States) at 14 and 21 days.

### RNA Isolation and Real-Time qPCR Analysis

After the cells were rinsed with PBS at 4°C, total mRNA was extracted using Trizol^®^ reagent and reverse transcribed into cDNA by PrimeScript^TM^ RT reagent Kit (RR037A). For miRNAs analysis, miRNA were reverse transcribed to cDNAs using the miRNA First-Strand cDNA Synthesis (Sangon, China) with the Stem-loop Method by primers (Supplementary Table 2). RT- qPCR was performed using SYBR^®^ Primix Ex Taq^TM^ by primers (Supplementary Table 3). U6 small nuclear RNA and GAPDH was used as an endogenous control for miRNA and mRNA. Relative differences in the PCR product amounts were evaluated by using the 2^–ΔΔ^CT method.

### Protein Extraction and Western Blot Analysis

Periodontal ligament stem cells were washed with cold PBS and lysed with RIPA containing 1% PMSF at 4°C for 30 min. Then cells were centrifuged at 4°C and 12,000 rpm for 30 min. For western blot analysis, 20 μg of each protein was separated on 10% SDS-polyacrylamide gels and transferred onto polyvinylidene difluoride membranes (Millipore, Bedford, MA, United States). The membranes were blocked with Tris-buffered saline containing 0.1% Tween-20 (TBST) and 5% fat-free milk, then incubated overnight at 4°C with primary antibodies against ALPL (1:1,000) (11187-1-AP), RUNX2 (1:1,000) (ab92336), SP7(1:1,000) (ab209484), COL1A1(1:500) (ab6308) and GAPDH (1:5,000). The membranes were washed three times with TBST and incubated with HRP-conjugated secondary antibodies (1:5,000) for 1 h at room temperature. The bands were visualized using enhanced chemiluminescence reagents (Millipore). The bands were measured using ImageJ software (National Institutes of Health, Bethesda, MD, United States).

### Statistical Analysis

Statistical analysis was performed using GraphPad prism 8. Student’s *t*-test or two-way ANOVA was used to analyze significant differences between groups. A *P*-value less than 0.05 was considered statistically significant. For biological replicates, three sets of cell-based assays were performed.

## Results

### Identification of Differentially Expressed Genes During Periodontal Ligament Stem Cells Osteogenic Differentiation

**FIGURE 1:**
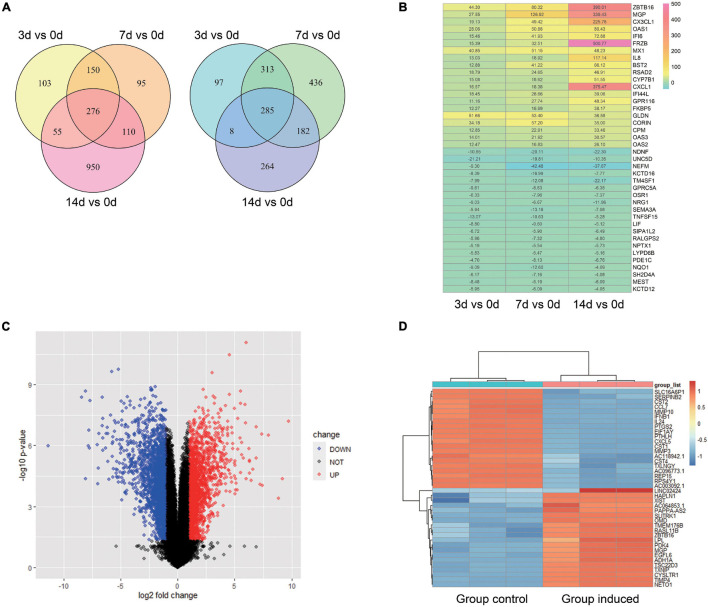
Analysis of differentially expressed genes (DEGs) of GSE99958 and GSE159507. For GSE99958, the CONORS package was used to analyze the differentially expressed genes (DEGs) between the Group induced 3, 7, 14 days and the Group control. A venn graph **(A)** shows the total 276 up-regulated and 285 down-regulated DEGs and a heat map **(B)** shows representative DEGs. The number in the heat map grid represents multiples of gene counts. For GSE159507, the limma package was used to analyze the difference genes between the Group induced and the Group control. A volcano plot **(C)** and heat map **(D)** reveal the 4,082 DEGs (total 1,908 up-regulated and 2,174 down-regulated DEGs). Red color indicates up-regulated genes, and blue indicates down-regulated genes. Group Control: PDLSCs treated with control medium. Group induced: PDLSCs treated with osteogenic medium.

**FIGURE 2:**
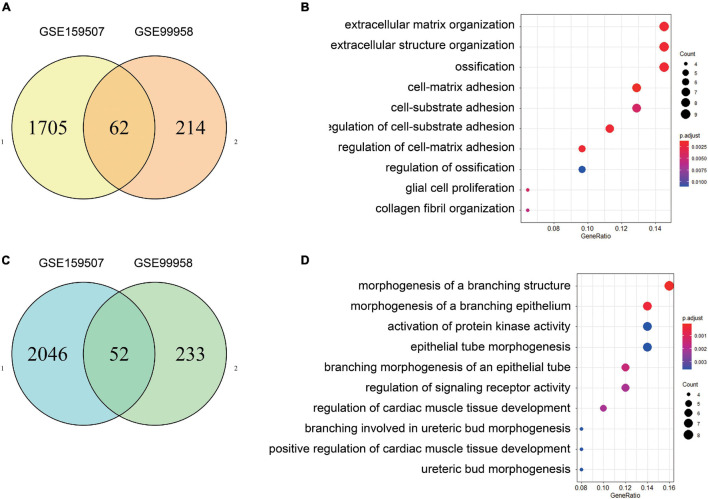
Identification and GO enrichment analysis of common differentially expressed genes. After the common DEGs of GSE99958 and GSE159507 were determined, there were 62 up-regulated genes and 52 down-regulated genes **(A,C)**. Functional enrichment analysis shows biological processes (BP), cellular components (CC), and molecular functions (MF) enrichment from analyses of the 62 up-regulated genes **(B)** and 52 down-regulated genes **(D)**. *P* value is 0.05. The GO enrichment analysis of up-regulated genes is mainly concentrated in “extracellular matrix organization,” “extracellular structure organization,” and “ossification.” The GO enrichment of down-regulated expressed genes mainly includes “morphogenesis of a branching structure,” “morphogenesis of a branching epithelium,” and “activation of protein kinase activity.”

For GSE99958, we used the package CORNAS (Low et al., 2017), which is specially used to analyze non-duplicate samples, and got 276 up-regulated genes and 285 down-regulated genes (Figure 1A). For GSE159507, the limma package was conducted to select 1,908 up-regulated genes and 2,174 down-regulated genes (Figure 1C). LogFC > 1 or LogFC < −1 and *P*-value < 0.05 were considered to indicate statistical significance. The representative DEGs are shown in a heat map (Figures 1B,D). Then 62 up-regulated genes and 52 down-regulated genes were identified by intersecting DEGs between GSE99958 and GSE159507 (Figures 2A,C).

### Gene Ontology and KEGG Enrichment Analyses of Different Expression miRNAs

Differentially expressed genes were separately enriched in GO terms, and the top most significant terms of up-regulated and down-regulated DEGs are shown (Figures 2B,D). Moreover, up-regulated DEGs were enriched in “extracellular matrix organization,” “extracellular structure organization,” and “ossification” Down-regulated DEGs were involved in “morphogenesis of a branching structure,” “morphogenesis of a branching epithelium,” and “activation of protein kinase activity.” The results of KEGG and detailed GO enrichment analyses of DEGs are provided in Supplementary Figure 2 and Supplementary Table 1.

### Protein-Protein Interaction Network Construction and Module Analysis

**FIGURE 3:**
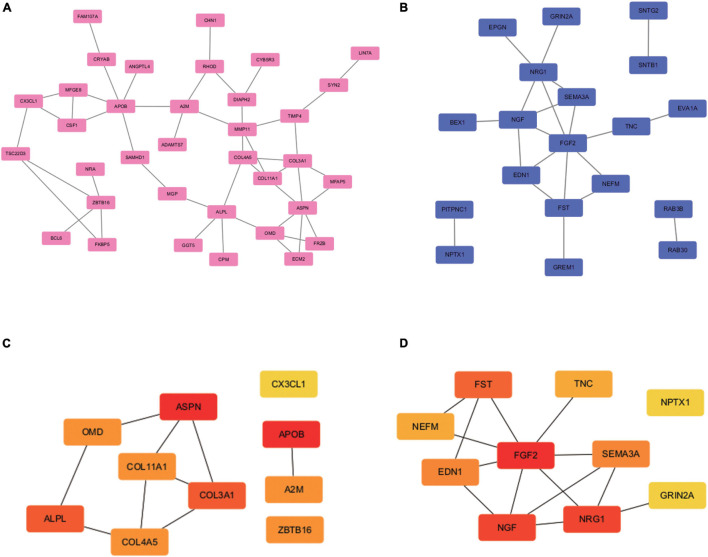
PPI network construction and hub genes screening. PPI network construction and module analysis **(A)** and **(B)** shows the PPI network of DEGs. The 62 up-regulated genes (35 nodes, 46 edges) are marked in red, while the 52 down-regulated genes (19 nodes, 21 edges) are marked in blue. The 10 up-regulated hub genes **(C)** and 10 down-regulated hub genes **(D)** were identified in the densest connected regions with the Degree algorithm, using cytoHubba. The score is indicated in red color. Darker color indicates a higher score.

The PPI network was analyzed using STRING (see text footnote 2). Analysis of functional interactions between 114 DEGs was performed in order to elucidate the mechanisms of osteogenic differentiation of PDLSCs. An interaction with a combined score was selected and used to construct a PPI network with Cytoscape software (Figures 3A,B). The top 10 up-regulated hub genes (APOB, ASPN, ALPL, COL3A1, A2M, COL4A5, OMD, COL11A1, ZBTB16, and CX3CL1 and down-regulated hub genes (FGF2, NGF, NRG1, FST, EDN1, SEMA3A, TNC, NEFM, GRIN2A, and NPTX1) were screened out separately through CytoHubba, a plug-in of Cytoscape (Figures 3C,D).

### Identification of Different Expression miRNAs During Periodontal Ligament Stem Cells Osteogenic Differentiation and miRNA-mRNA Network Prediction

**FIGURE 4:**
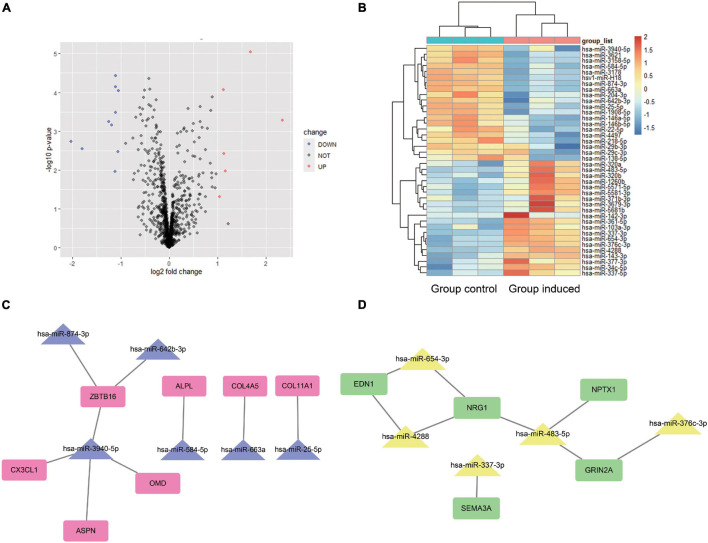
Differentially expressed miRNA analysis and miRNA-mRNA potential interaction network construction. For the miRNA-seq dataset GSE159508, limma package was used to analyze the DEMs between the Group induced and the Group Control. A volcano plot **(A)** and heat map **(B)** shows the 16 DEMs. Red color indicates six up-regulated miRNA, and blue indicates 10 down-regulated miRNA. The predicted target genes of DEMs is calculated by miRWalk website. After the predicted target genes of DEMs and the obtained hub genes were intersected, a negatively regulated PPI of miRNA-mRNA network was obtained **(C,D)**. We obtained five up-regulated (miR-337-3p, miR-376c-3p, miR-4288, miR-483-5p, and miR-654-3p) and 6 down-regulated (miR-25-5p, miR-3940-5p, miR-584-5p, miR-642b-3p, miR-663a, and miR-874-3p) DEMs that potentially interacted to hub genes. Group Control: PDLSCs treated with control medium. Group induced: PDLSCs treated with osteogenic medium.

For GSE159508, we used the limma package to obtain six up-regulated miRNAs (LogFC > 1 and *P*-value < 0.05) and 10 down-regulated miRNAs (LogFC < −1 and *P*-value < 0.05) (Figures 4A,B). Using the miRwalk website (see text footnote 4), the predicted target genes of DEMs were determined. After the predicted target genes of DEMs and the obtained hub genes were intersected, a negatively regulated PPI network of miRNA-mRNA was obtained (Figures 4C,D). We obtained five up-regulated (miR-337-3p, miR-376c-3p, miR-4288, miR-483-5p, and miR-654-3p) and 6 down-regulated (miR-25-5p, miR-3940-5p, miR-584-5p, miR-642b-3p, miR-663a, and miR-874-3p) DEMs that potentially interacted to hub genes.

### Hub Genes and Key Different Expression miRNAs Change During the Osteogenic Differentiation of Periodontal Ligament Stem Cells by qPCR

**FIGURE 5:**
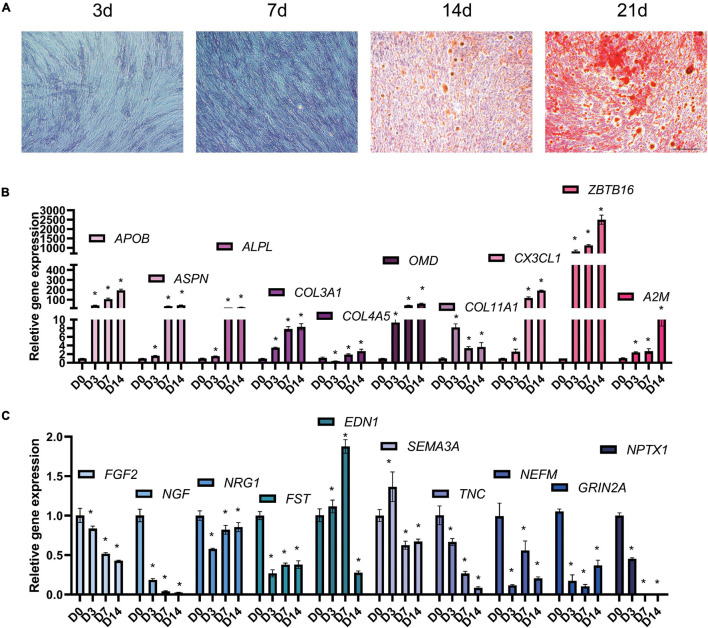
Hub genes change during osteogenic differentiation of PDLSCs *in vitro*. PDLSCs cells were cultured in osteogenic induction medium for 0, 3, 7, 14 days and tested by qPCR. **(A)** Representative microscope pictures show results of ALP staining at 3 and 7 days, and the results of Alizarin Red staining at 14 and 21 days. ALP and ARS staining were used to prove that the process of osteogenic differentiation was successful. **(B)** qPCR results shows up-regulated hub genes APOB, ASPN, ALPL, COL3A1, A2M, COL4A5, OMD, COL11A1, ZBTB16, and CX3CL1 **(C)** down-regulated hub genes FGF2, NGF, NRG1, FST, EDN1, SEMA3A, TNC, NEFM, GRIN2A, and NPTX1 during osteogenic differentiation of PDLSCs. Bar = 25 μm. *p < 0.05.

**FIGURE 6:**
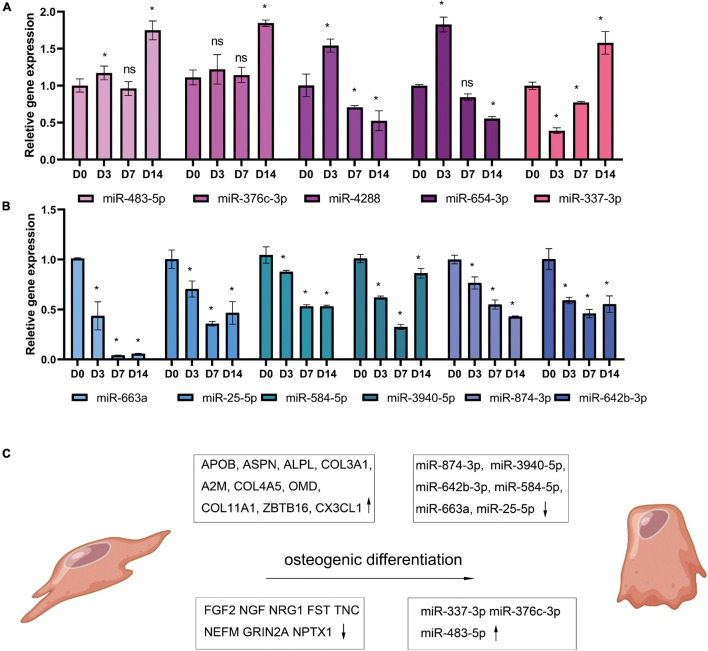
Candidate miRNAs change during osteogenic differentiation of PDLSCs *in vitro*. PDLSCs cells were cultured in osteogenic induction medium for 0, 3, 7, 14 days and reverse transcribed with Stem-loop Method and then tested by qPCR. The histogram shows qPCR results of five up-regulated miRNAs **(A)** and 6 down-regulated miRNAs **(B)**. Demonstration diagram of hub genes and miRNAs change patterns verified during PDLSCs osteogenic differentiation *in vitro* **(C)**. *p < 0.05.

Periodontal ligament stem cells were isolated, passaged, and identified (Supplementary Figure 1). At 3, 7, 14, and 21 days after osteogenic differentiation, ALP and ARS staining confirmed the osteogenic differentiation of PDLSCs (Figure 5A). The result of qPCR (Figures 5B,C) shows that the expression of 10 up-regulated DEGs (ALPL, COL4A5, COL3A1, COL11A, OMD, APOB, ZBTB16, ASPN, CX3CL, and A2M) and 10 down-regulated DEGs (NEFM, FST, TNC, NPTX1, EDN1, FGF2, SEMA3A, NGF, NRG1, and GRIN2A). The qPCR results of miRNA reveals that the expression of 6 down-regulated DEMs (miR-25-5p, miR-3940-5p, miR-584-5p, miR-642b-3p, miR-663a, and miR-874-3p) and three up-regulated DEMs (miR-337-3p, miR-376c-3p, and miR-483-5p) was consistent with the analysis results of miRNA-seq (Figures 6A,B). The expression of miR-4288 and miR-654-3p were increased at day 3 and decreased at day 7 and day 14 during osteogenic differentiation. The possible change patterns of hub genes and miRNAs verified during PDLSCs osteogenic differentiation in vitro are shown in a schematic illustration (Figure 6C).

### miR-584-5p Regulates the Osteogenic Differentiation of Periodontal Ligament Stem Cells

**FIGURE 7:**
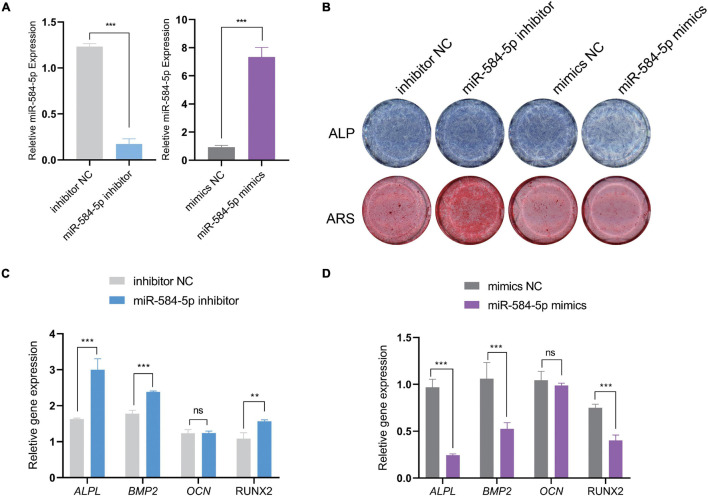
MiR-584-5p regulates the osteogenic differentiation of PDLSCs *in vitro*. PDLSCs were transfected with inhibitor NC, miR584-5p inhibitor, mimics NC and miR584-5p mimics. After 24 h of transfection, the relative expression of miR584-5p was detected by qPCR **(A)**. Cells were cultured in osteogenic induction medium for 7, 14 days. ALP staining (7 days) and Alizarin Red staining (14 days) were used to detect ALP activity and the formation of mineralized nodules, respectively **(B)**. Osteogenesis related indicators ALPL, BMP2, RUNX2 and OCN were used to further prove the osteogenic regulation of miR-584-5p **(C,D)**. **p < 0.01 and ***p < 0.001.

**FIGURE 8:**
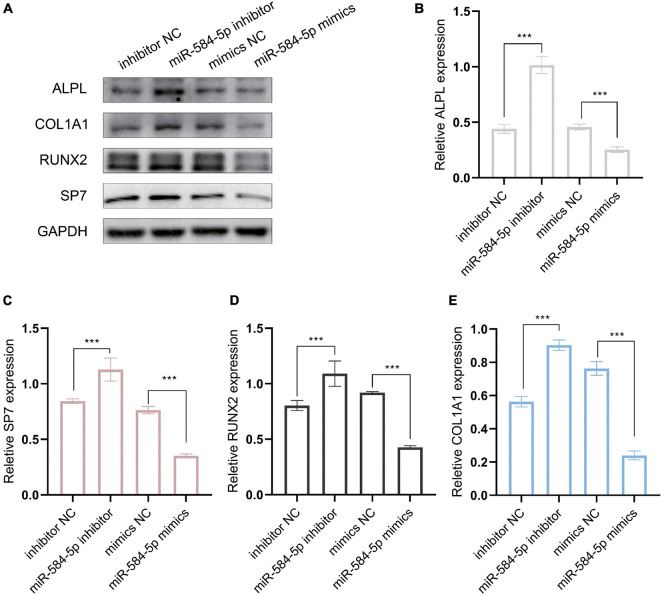
MiR-584-5p regulates osteogenic differentiation-related protein expression *in vitro*. **(A)** The osteogenic differentiation-related protein expression was detected by WB assay in different groups at day 7. The semi-quantitative results of related proteins are calculated and displayed, ALPL **(B)**, SP7 **(C)**, RUNX2 **(D)**, and COL1A1 **(E)**. ***p < 0.001.

We predict that miR584-5p can bind to ALPL and may regulate the expression of ALPL. ALPL is a very important osteogenic regulatory gene. Tian et al. (2020) found that inhibiting miR-584-5p in dental pulp stem cells can promote cell proliferation. Therefore, we chose miR-584-5p for follow-up experiments. In order to verify the regulation of miR-584-5p on the osteogenic differentiation of PDLSCs. PDLSCs were transfected with inhibitor NC, miR584-5p inhibitor, mimics NC and miR584-5p mimics. MiR-584-5p inhibitor or miR-584-5p mimics effectively reduced or increased the expression of miR-584-5p, respectively (Figure 7A). Cells were cultured in the osteogenic induction medium for 7, 14 days. ALP activity and the formation of mineralized nodules, respectively, were detected by ALP staining and Alizarin red staining (Figure 7B). Osteogenesis-related indicators ALPL, BMP2, RUNX2, and OCN were used to further prove the osteogenic regulation of miR-584-5p (Figures 7C,D). The osteogenic differentiation-related protein expression was detected by WB assay in different groups at day 7 (Figure 8A). The semi-quantitative results of related proteins are calculated and displayed, ALPL, SP7, RUNX2, and COL1A1 (Figures 8B–E).

## Discussion

In this study, through the DEGs analysis of multiple datasets, the gene changes of PDLSCs after osteoinduction were obtained, and the GO enrichment analysis of DEGs was performed. Fan reported (Fan et al., 2020) that the main enrichment pathways of BMSCs osteogenic differentiation were extracellular matrix organization, ossification, negative regulation of cell proliferation, vasculature development, cell division and spindle. Wang reported (Yang et al., 2019) that regulation of the cellular amino acid metabolic process, DNA metabolic process and regulation of apoptotic process were mostly enriched during the osteogenic differentiation of BMSCs. Some pathways obtained in this study overlap with the enrichment pathways of BMSCs. Our study found that extracellular matrix organization, extracellular structure organization and ossification played an important role in the osteogenic differentiation of PDLSCs. The process of growth and osteogenic differentiation of pluripotent stem cells mainly includes cell adhesion, proliferation, extracellular matrix secretion and maturation, and extracellular matrix mineralization (Stein et al., 2004). The enriched function pathways derived from the analysis of these DEGs can well meet the functional requirements needed in the process of osteogenic differentiation.

A total of 114 DEGs were screened in the two datasets, and 62 up-regulated genes and 52 down-regulated genes were identified. Then, 10 up-regulated hub genes and 10 down-regulated hub genes were screened out through Cytoscape. Among these 10 up-regulated hub genes, most of them such as ALPL, COL4A5 (Sun J. et al., 2021), COL3A1 (Minaříková et al., 2015; Williams et al., 2018), COL11A1 and OMD (Lin et al., 2019, 2021; Ustriyana et al., 2021) are reported to be related to bone mineralization. This result partly proves that DEGs analysis is effective. Other up-regulated hub genes are APOB, ZBTB16, ASPN, CX3CL1, and A2M. ZBTB16 has been reported to be significantly elevated in the osteogenic differentiation of human mesenchymal stem cells (MSCs) (Ikeda et al., 2005), and promote the osteogenic differentiation of dental follicle–derived precursor cells (DFCs), another type of odontogenic pluripotent stem cells (Felthaus et al., 2014). APOB gene is related to the regulation of lipid metabolism (Seo et al., 2004), and there is no literature report that it is directly involved in the regulation of osteogenic differentiation. In addition, APOB was also strongly upregulated in the osteogenic differentiation of canine dental pulp stem cells (Sirirat et al., 2020).

Several studies point out that ASPN was increased in the osteogenic differentiation of PDLSCs (Yamada et al., 2006; Ueda et al., 2016; Garna et al., 2020), and ASPN is a specific molecule marker of PDLSCs. However, overexpression of ASPN in PDLSCs alone cannot promote the osteogenic differentiation of PDLSCs, but inhibits the osteogenic differentiation of PDLSCs partly (Yamada et al., 2007). In addition, ASPN accelerated bone resorption at the orthodontic tension side (Zhang et al., 2020). This contradictory result may be related to the different subgroups of PDLSCs, or to the balance of bone formation and resorption. The molecular mechanism and effects of ASPN in the osteogenic differentiation of PDLSCs needs more research.

The expression of osteoblasts CX3CL1 is critical for osteoclast differentiation. CX3CL1 plays an important role in bone formation and resorption balance by playing dual functions as a chemotactic factor and adhesion molecule for osteoclast precursors expressing CX3CR1 (Koizumi et al., 2009; Xiao et al., 2009; Hoshino et al., 2013). This study found that the expression of CX3CL1 increased during the osteogenic differentiation of PDLSCs. There are two possible explanations, PDLSCs express CX3CL1 that may allow chemotactic migration of osteoclasts to bone remodeling area. Another possibility is PDLSCs which also express the receptor CX3CR1, and secreted CX3CL1 can homing more PDLSCs even BMSCs (Zhang et al., 2015) to the position of bone remodeling through paracrine. The specific effects of CX3CL1 during the osteogenic differentiation of PDLSCs has not been elucidated, and the CX3CL1-CX3CR1 axis may serve as a potential chemokine pathway in order to improve the therapeutic efficacy of these cells.

A recent study (Ghale-Noie et al., 2018) reported that serum protein level of A2M was raised in avascular necrosis of the femoral head patients. A2M also has several roles in fibrinolysis and the coagulation cascade (de Boer et al., 1993). Sadeghi showed that ACTH promoted the osteogenic differentiation of MSCs by up-regulation of A2M (Sadeghi et al., 2020).

Down-regulated 10 hub genes was studied and analyzed by searching the published literature. NEFM, GRIN2A, NPTX1, NRG1, and NGF genes are related to nerve cell development and nerve signal transmission. NEFM is associated with neural maturation (Urrutia et al., 2019; Kawase-Koga et al., 2020) and is down-regulated after early adipogenic differentiation (Urrutia et al., 2019; Marcon et al., 2020; Sadeghi et al., 2020; Nie et al., 2021). GRIN2A is found in nerve cells (neurons) in the brain and spinal cord and one component of a subset of NMDA receptors. NPTX1 may be involved in mediating the uptake of synaptic material during synapse remodeling. Chorion Stromal Cells express higher NPTX1 than Amnion Stromal Cells (Jones et al., 2021). NRG1 is one of four proteins in the neuregulin family that act on the EGFR family of receptors. It was decreased during BMSCs chondrocyte differentiation (Maumus et al., 2020). Nerve growth factor (NGF) is a neurotrophic factor and neuropeptide primarily and was reported to mediate osteogenic differentiation of BMSCs (Peyton, 2017). Interestingly, PDLSCs were able to induce neural progenitor differentiation.

FST was decreased during DPSCs osteogenic differentiation [62] and FST did not enhance osteogenic differentiation of MSCs, but increased committed osteoblast mineralization (Fahmy-Garcia et al., 2019). TNC, as an Extracellular protein, helps osteoblast adhesion to the ECM by creating cell-matrix adhesion sites (Baroncelli et al., 2018). TNC is regarded as one of the markers of the tendon extracellular matrix (Liu et al., 2018). EDN1 is a potent vasoconstrictor and produced by vascular endothelial cells. EDN-1 overexpressed BMSCs showed increased proliferation and significantly increased osteogenesis potential ability (Hu et al., 2017). The overexpression of EDN1 in ADSCs significantly promoted the proliferation and migration of co-cultured HUVECs (Wang et al., 2021). FGF2 inhibited the osteogenic differentiation of PDLSCs. For BMSCs, FGF2 has no obvious function in improving the osteogenic-related genes, but it can ameliorate the impaired osteogenesis (Hao et al., 2020). However, some authors have reported different results. FGF2 enhances the proliferation and osteogenic differentiation of MSCs to promote bone formation (Li et al., 2017; Poudel et al., 2019). Autoregulation of osteocyte SEMA3A promotes bone formation and counteracts bone aging (Hayashi et al., 2019) and is a factor that accelerates osteogenic differentiation (Qiao et al., 2018; Sun Z. et al., 2021). In summary, the effects of FST, TNC, EDN1, FGF2, and SEMA3A in the osteogenic differentiation of PDLSCs need more research and work.

It has been reported that miRNA can regulate the osteogenic differentiation of BMSCs and PDLSCs. For BMSC, MiRNAs control gene expression in osteogenic differentiation by regulating two crucial signaling cascades in osteogenesis: the transforming growth factor-beta (TGF-β)/bone morphogenic protein (BMP) and the Wingless/Int-1(Wnt)/β- catenin signaling pathways (Mazziotta et al., 2021). In previous literature, miR-17, miR-21, miR23a, miR-24, miR-132, miR-138, miR-214, miR-218, miR-543, and miR1305 have been reported to regulate PDLSCs Osteogenic differentiation (Ahmad et al., 2021). In these documents, the target genes of miRNA are mostly concentrated in the WNT signaling pathway, such as TCF3 (Liu et al., 2013), CTNNB1 (Cao et al., 2017), or the TGF-β pathway (Wei et al., 2015). In addition, miR-1305 (Chen and Liu, 2017) and miR-218 (Gay et al., 2014) have been reported to directly regulate the expression of RUNX2. The research results of previous scholars provide us with ideas for further searching for the target genes of DEMs. The miRNA-seq analysis obtained 11 DEMs. The increased miR-654-3p, miR-4288 and decreased miR-663a and miR-874-3p have the same trends as reported in the literature during the osteogenic differentiation of PDLSCs (Hao et al., 2017) (GSE106887). In addition, miR-584-5p was also reduced after osteogenic differentiation of DPSCs (Liu et al., 2020). MiR-337-3p upregulated in DPSCs while downregulated in BMSCs during osteogenic differentiation (Gaus et al., 2021). The role and expression trend of these miRNAs are consistent with our analysis results. However, MiR-483–5p is involved in the pathogenesis of osteoporosis by promoting osteoclast differentiation (Li et al., 2020). MiR-376c-3p inhibits BMSCs osteogenesis (Camp et al., 2018; Kureel et al., 2018). The role of these two miRNAs needs further study. There is no literature describing miR-25-5p, miR-642b-3p, and miR-3940-5p directly related to osteogenesis.

The sample size of the datasets we included is not very large. In addition, we only selected miR-584-5p as an in-depth study and did not prove the exact target gene of miR-584. This is the shortcoming of our experiment. In this study, through the analysis of multiple datasets, the expression changes of nine miRNAs and 18 hub genes were obtained. This provides new preliminary target genes for studying the osteogenic differentiation of PDLSCs.

## Data Availability Statement

The datasets GSE99958, GSE159507, and GSE159508 for this study can be found in the (Gene Expression Omnibus) (https://www.ncbi.nlm.nih.gov/geo/query/acc.cgi).

## Author Contributions

CW, LD, ZJ, JZ, and YW contributed in the material preparation, collected and analyzed the data. CW and GY wrote the first draft of the manuscript. All authors contributed to the study conception and design, commented on previous versions of the manuscript, and read and approved the final manuscript.

## Conflict of Interest

The authors declare that the research was conducted in the absence of any commercial or financial relationships that could be construed as a potential conflict of interest.

## Publisher’s Note

All claims expressed in this article are solely those of the authors and do not necessarily represent those of their affiliated organizations, or those of the publisher, the editors and the reviewers. Any product that may be evaluated in this article, or claim that may be made by its manufacturer, is not guaranteed or endorsed by the publisher.

## Footnotes

1
http://www.ncbi.nlm.nih.gov/geo/

2
https://string-db.org/

3
http://www.cytoscape.org/download.php

4
http://mirwalk.umm.uni-heidelberg.de/

## Supplementary Material

Supplementary Figure 1 Isolation and characterization of human PDLSCs. PDLSCs at P0 showed a typical fibroblast-like spindle appearance **(A)**. Mineralized nodules were stained by ARS at 14d **(B)**, cartilage tissue was stained by Alcian Blue at 21d **(C)**, Clone formation **(D)**, and lipid droplets were stained by Oil Red O staining at 14d **(E)**.

Supplementary Figure 2 The GOCord circle graph and KEGG enrichment analysis of DEGs. The GOCord circle graph shows the relationship between the GO terms and the up-regulated 62 DEGs **(A)** or the 52 down-regulated DEGs **(B)**. Each GO term is indicated with a specific color. The **(C)** and **(D)** show KEGG enrichment analysis of DEGs. The KEGG enrichment analysis of up-regulated genes **(C)** is mainly concentrated in “Protein digestion and absorption” and “Thiamine metabolism.” The KEGG enrichment of down-regulated expressed genes **(D)** mainly includes the “Calcium signaling pathway” and “TGF-beta signaling pathway.”

Supplementary Table 1 Detailed GO enrichment analyses of DEGs.

Supplementary Table 2 MiRNA primers used in this study.

Supplementary Table 3 Hub genes primers used in this study.

## Abbreviations

BMSCs: bone marrow-derived mesenchymal stem cells
MSC: mesenchymal stem cell
IBMX: 3-isobutyl-1-methyl-xanthine
α-MEM: alpha Modification Minimum Essential Medium Eagle
FBS: fetal bovine serum
ARS: alizarin red staining
PDLSCs: periodontal ligament stem cells
ADSCs: adipose-derived stem cells
DEMs: differentially expressed miRNAs
DEGs: differentially expressed genes
GEO: Gene Expression Omnibus
PDLCs: periodontal ligament cells
GO: gene ontology
PPI: Protein-protein interaction network
qPCR: quantitative Polymerase Chain Reaction
DFCs: dental follicle derived precursor cells.

## References

[B1] Abdan A. Mustafa A. Kathrin A. A. Opeyemi A. R. Filipe A. d. C. Collins A. (2020) *Analyse der Genexpression von Humanen Stro-1-Positiven Zahnkeim- und Beckenkammzellen in DME-Medium und Osteogenem Differenzierungsmedium*. Doctoral thesis. Available online at: https://ediss.uni-goettingen.de/handle/21.11130/00-1735-0000-0005-145D-D?show=full (accessed August 18, 2020).

[B2] Ahmad P. Stoddart M. J. Della Bella E. (2021). The role of noncoding RNAs in osteogenic differentiation of human periodontal ligament stem cells. *Craniomaxillof. Traum. Reconstr. Open* 6:247275122199922.

[B3] Aprilianti N. A. Rahmadhani D. Rizqianti Y. Ridwan R. D. Ramadhani N. F. Nugraha A. P. (2020). Periodontal ligament stem cells, solcoseryl pasta incoporated nano-hydroxyapatite silica gel scaffold for bone defect regeneration in chronic periodontitis: a review actinomycetemcomitans. *Biochem. Cell. Arch.* 20 3101–3106.

[B4] Baroncelli M. van der Eerden B. C. J. Chatterji S. Rull Trinidad E. Kan Y. Y. Koedam M. (2018). Human osteoblast-derived extracellular matrix with high homology to bone proteome is osteopromotive. *Tissue Eng. Part A* 24 1377–1389. 10.1089/ten.tea.2017.0448 29667532

[B5] Bini L. Schvartz D. Carnemolla C. Besio R. Garibaldi N. Sanchez J. (2021). Intracellular and extracellular markers of lethality in osteogenesis imperfecta: a quantitative proteomic approach. *Int. J. Mol. Sci.* 22:429. 10.3390/ijms22010429 33406681 PMC7795927

[B6] Camp E. Pribadi C. Anderson P. J. Zannettino A. C. W. Gronthos S. (2018). miRNA-376c-3p mediates TWIST-1 inhibition of bone marrow-derived stromal cell osteogenesis and can reduce aberrant bone formation of TWIST-1 haploinsufficient calvarial cells. *Stem Cells Dev.* 27 1621–1633. 10.1089/scd.2018.0083 30229694

[B7] Cao F. Zhan J. Chen X. Zhang K. Lai R. Feng Z. (2017). miR-214 promotes periodontal ligament stem cell osteoblastic differentiation by modulating Wnt/betacatenin signaling. *Mol. Med. Rep.* 16 9301–9308. 10.3892/mmr.2017.7821 29152645 PMC5779983

[B8] Chen Z. Liu H. (2017). Restoration of miR-1305 relieves the inhibitory effect of nicotine on periodontal ligament-derived stem cell proliferation, migration, and osteogenic differentiation. *J. Oral Pathol. Med.* 46 313–320. 10.1111/jop.12492 27604968

[B9] de Boer J. P. Creasey A. A. Chang A. Abbink J. J. Roem D. Eerenberg A. J. (1993). Alpha-2-macroglobulin functions as an inhibitor of fibrinolytic, clotting, and neutrophilic proteinases in sepsis: studies using a baboon model. *Infect. Immun.* 61 5035–5043. 10.1128/iai.61.12.5035-5043.1993 7693593 PMC281280

[B10] Fahmy-Garcia S. Farrell E. Witte-Bouma J. Robbesom-van Den Berge I. Suarez M. Mumcuoglu D. (2019). Follistatin effects in migration, vascularization, and osteogenesis in vitro and bone repair in vivo. *Front. Bioeng. Biotechnol.* 7:38. 10.3389/fbioe.2019.00038 30881954 PMC6405513

[B11] Fan T. Qu R. Yu Q. Sun B. Jiang X. Yang Y. (2020). Bioinformatics analysis of the biological changes involved in the osteogenic differentiation of human mesenchymal stem cells. *J. Cell. Mol. Med.* 24 7968–7978. 10.1111/jcmm.15429 32463168 PMC7348183

[B12] Felthaus O. Gosau M. Morsczeck C. (2014). ZBTB16 induces osteogenic differentiation marker genes in dental follicle cells independent from RUNX2. *J. Periodontol.* 85 e144–e151. 24359167 10.1902/jop.2013.130445

[B13] Garna D. Kaur M. Hughes F. J. Ghuman M. (2020). Comparison of the expression of periodontal markers in dental and bone marrow-derived mesenchymal stem cells. *Open Dentist. J.* 14 196–202. 10.2174/1874210602014010196

[B14] Gaus S. Li H. Li S. Wang Q. Kottek T. Hahnel S. (2021). Shared genetic and epigenetic mechanisms between the osteogenic differentiation of dental pulp stem cells and bone marrow stem cells. *Biomed. Res. Int.* 2021 1–25. 10.1155/2021/6697810 33628811 PMC7884974

[B15] Gay I. Cavender A. Peto D. Sun Z. Speer A. Cao H. (2014). Differentiation of human dental stem cells reveals a role for microRNA-218. *J. Periodontal Res.* 49 110–120. 10.1111/jre.12086 23662917 PMC3758777

[B16] Ghale-Noie Z. N. Hassani M. Kachooei A. R. Kerachian M. A. (2018). High serum alpha-2-macroglobulin level in patients with osteonecrosis of the femoral head. *Arch. Bone Joint Surg.* 6:219. PMC5990713 29911139

[B17] Gu X. Li M. Jin Y. Liu D. Wei F. (2017). Identification and integrated analysis of differentially expressed lncRNAs and circRNAs reveal the potential ceRNA networks during PDLSC osteogenic differentiation. *BMC Genet.* 18:100. 10.1186/s12863-017-0569-4 29197342 PMC5712120

[B18] Hao Y. Ge Y. Li J. Hu Y. Wu B. Fang F. (2017). Identification of MicroRNAs by microarray analysis and prediction of target genes involved in osteogenic differentiation of human periodontal ligament stem cells. *J. Periodontol.* 88 1105–1113. 10.1902/jop.2017.170079 28598283

[B19] Hao Y. Wu M. Wang J. (2020). Fibroblast growth factor-2 ameliorates tumor necrosis factor-alpha-induced osteogenic damage of human bone mesenchymal stem cells by improving oxidative phosphorylation. *Mol. Cell Probe* 52:101538. 10.1016/j.mcp.2020.101538 32084581

[B20] Hayashi M. Nakashima T. Yoshimura N. Okamoto K. Tanaka S. Takayanagi H. (2019). Autoregulation of osteocyte Sema3A orchestrates estrogen action and counteracts bone aging. *Cell Metab.* 29 627.e4–637.e4. 30661929 10.1016/j.cmet.2018.12.021

[B21] Hoshino A. Ueha S. Hanada S. Imai T. Ito M. Yamamoto K. (2013). Roles of chemokine receptor CX3CR1 in maintaining murine bone homeostasis through the regulation of both osteoblasts and osteoclasts. *J. Cell Sci.* 126(Pt 4) 1032–1045. 23264747 10.1242/jcs.113910

[B22] Hu L. Liu Y. Wang S. (2018). Stem cell-based tooth and periodontal regeneration. *Oral Dis.* 24 696–705. 10.1111/odi.12703 28636235

[B23] Hu L. Wang X. Jiang X. Xu L. Pan H. (2017). In vivo and in vitro study of osteogenic potency of endothelin-1 on bone marrow-derived mesenchymal stem cells. *Exp. Cell Res.* 357 25–32. 10.1016/j.yexcr.2017.04.018 28432001

[B24] Ikeda R. Yoshida K. Tsukahara S. Sakamoto Y. Tanaka H. Furukawa K. (2005). The promyelotic leukemia zinc finger promotes osteoblastic differentiation of human mesenchymal stem cells as an upstream regulator of CBFA1. *J. Biol. Chem.* 280 8523–8530. 10.1074/jbc.m409442200 15623533

[B25] Iwasaki K. Washio K. Meinzer W. Tsumanuma Y. Yano K. Ishikawa I. (2019). Application of cell-sheet engineering for new formation of cementum around dental implants. *Heliyon* 5:e01991. 10.1016/j.heliyon.2019.e01991 31338459 PMC6626299

[B26] Jones B. Li C. Park M. S. Lerch A. Jacob V. Johnson N. (2021). Comprehensive comparison of amnion stromal cells and chorion stromal cells by RNA-Seq. *Int. J. Mol. Sci.* 22:1901. 10.3390/ijms22041901 33672986 PMC7918623

[B27] Kämmerer P. W. Scholz M. Baudisch M. Liese J. Wegner K. Frerich B. (2017). Guided bone regeneration using collagen scaffolds, growth factors, and periodontal ligament stem cells for treatment of peri-implant bone defects in vivo. *Stem Cells Int.* 2017:3548435. 10.1155/2017/3548435 PMC5603746 28951742

[B28] Kawase-Koga Y. Fujii Y. Yamakawa D. Sato M. Chikazu D. (2020). Identification of neurospheres generated from human dental pulp stem cells in xeno-/serum-free conditions. *Regen. Ther.* 14 128–135. 10.1016/j.reth.2019.11.006 32099873 PMC7029376

[B29] Koizumi K. Saitoh Y. Minami T. Takeno N. Tsuneyama K. Miyahara T. (2009). Role of CX3CL1/fractalkine in osteoclast differentiation and bone resorption. *J. Immunol.* 183 7825–7831. 10.4049/jimmunol.0803627 19923448

[B30] Kureel J. John A. A. Prakash R. Singh D. (2018). MiR 376c inhibits osteoblastogenesis by targeting Wnt3 and ARF-GEF-1 -facilitated augmentation of beta-catenin transactivation. *J. Cell Biochem.* 119 3293–3303. 10.1002/jcb.26490 29125885

[B31] Lee J. Kim E. Han S. Kang K. L. Heo J. S. (2017). Evaluating the oxysterol combination of 22 (S)-hydroxycholesterol and 20 (S)-hydroxycholesterol in periodontal regeneration using periodontal ligament stem cells and alveolar bone healing models. *Stem Cell Res. Ther.* 8 1–12. 29208033 10.1186/s13287-017-0725-9 PMC5717822

[B32] Lee J. S. Lee J. B. Cha J. K. Choi E. Y. Park S. Y. Cho K. S. (2017). Chemokine in inflamed periodontal tissues activates healthy periodontal ligament stem cell migration. *J. Clin. Periodontol.* 44 530–539. 10.1111/jcpe.12710 28207939

[B33] Li K. Chen S. Cai P. Chen K. Li L. Yang X. (2020). MiRNA-483–5p is involved in the pathogenesis of osteoporosis by promoting osteoclast differentiation. *Mol. Cell Probe* 49:101479. 10.1016/j.mcp.2019.101479 31706013

[B34] Li L. Qi Q. Luo J. Huang S. Ling Z. Gao M. (2017). FOXO1-suppressed miR-424 regulates the proliferation and osteogenic differentiation of MSCs by targeting FGF2 under oxidative stress. *Sci. Rep. UK* 7:42331. 10.1038/srep42331 PMC5301230 28186136

[B35] Lin W. Gao L. Jiang W. Niu C. Yuan K. Hu X. (2019). The role of osteomodulin on osteo/odontogenic differentiation in human dental pulp stem cells. *BMC Oral Health* 19:22. 10.1186/s12903-018-0680-6 30670012 PMC6341608

[B36] Lin W. Zhu X. Gao L. Mao M. Gao D. Huang Z. (2021). Osteomodulin positively regulates osteogenesis through interaction with BMP2. *Cell Death Dis.* 12 1–13. 33542209 10.1038/s41419-021-03404-5 PMC7862363

[B37] Liu J. Chen B. Bao J. Zhang Y. Lei L. Yan F. (2019a). Macrophage polarization in periodontal ligament stem cells enhanced periodontal regeneration. *Stem Cell Res. Ther.* 10 1–11. 31730019 10.1186/s13287-019-1409-4 PMC6858751

[B38] Liu J. Ruan J. Weir M. D. Ren K. Schneider A. Wang P. (2019b). Periodontal bone-ligament-cementum regeneration via scaffolds and stem cells. *Cells Basel* 8:537. 10.3390/cells8060537 31167434 PMC6628570

[B39] Liu Q. Zhu Y. Amadio P. C. Moran S. L. Gingery A. Zhao C. (2018). Isolation and characterization of multipotent turkey tendon-derived stem cells. *Stem Cells Int.* 2018 1–10. 10.1155/2018/3697971 29977306 PMC6011053

[B40] Liu W. Liu Y. Guo T. Hu C. Luo H. Zhang L. (2013). TCF3, a novel positive regulator of osteogenesis, plays a crucial role in miR-17 modulating the diverse effect of canonical Wnt signaling in different microenvironments. *Cell Death Dis.* 4:e539. 10.1038/cddis.2013.65 23492770 PMC3613843

[B41] Liu Z. Xu S. Dao J. Gan Z. Zeng X. (2020). Differential expression of lncRNA/miRNA/mRNA and their related functional networks during the osteogenic/odontogenic differentiation of dental pulp stem cells. *J. Cell Physiol.* 235 3350–3361. 10.1002/jcp.29223 31549394 PMC13482696

[B42] Low J. Khang T. F. Tammi M. T. (2017). CORNAS: coverage-dependent RNA-Seq analysis of gene expression data without biological replicates. *BMC Bioinformatics* 18(Suppl. 16):575. 10.1186/s12859-017-1974-4 29297307 PMC5751784

[B43] Ma Y. Ji Y. Zhong T. Wan W. Yang Q. Li A. (2017). Bioprinting-based PDLSC-ECM screening for in vivo repair of alveolar bone defect using cell-laden, injectable and photocrosslinkable hydrogels. *ACS Biomater. Sci. Eng.* 3 3534–3545. 10.1021/acsbiomaterials.7b00601 33445388

[B44] Marcon B. H. Spangenberg L. Bonilauri B. Robert A. W. Angulski A. B. B. Cabo G. C. (2020). Data describing the experimental design and quality control of RNA-Seq of human adipose-derived stem cells undergoing early adipogenesis and osteogenesis. *Data Brief* 28:105053. 10.1016/j.dib.2019.105053 31989002 PMC6970145

[B45] Maumus M. Fonteneau G. Ruiz M. Assou S. Boukhaddaoui H. Pastoureau P. (2020). Neuromedin B promotes chondrocyte differentiation of mesenchymal stromal cells via calcineurin and calcium signaling. *bioRvix* [Preprint]. 10.21203/rs.3.rs-52312/v1 PMC8525028 34663442

[B46] Mazziotta C. Lanzillotti C. Iaquinta M. R. Taraballi F. Torreggiani E. Rotondo J. C. (2021). MicroRNAs modulate signaling pathways in osteogenic differentiation of mesenchymal stem cells. *Int. J. Mol. Sci.* 22:2362. 10.3390/ijms22052362 33673409 PMC7956574

[B47] Minaříková M. Oralová V. Veselá B. Radlanski R. J. Matalová E. (2015). Osteogenic profile of mesenchymal cell populations contributing to alveolar bone formation. *Cells Tissues Organs* 200 339–348. 10.1159/000439165 26451912

[B48] Nagata M. Iwasaki K. Akazawa K. Komaki M. Yokoyama N. Izumi Y. (2017). Conditioned medium from periodontal ligament stem cells enhances periodontal regeneration. *Tissue Eng. Part A* 23 367–377. 10.1089/ten.tea.2016.0274 28027709 PMC5444511

[B49] Nie F. Bi H. Zhang C. Ding P. (2021). Differentiation potential and mRNA profiles of human dedifferentiated adipose cells and&nbsp;adiposederived stem cells from young donors. *Mol. Med. Rep.* 23:47. 10.3892/mmr.2020.11685 PMC7705993 33200799

[B50] Peyton S. G. F. (2017). *The Molecular Regulation of Nerve Growth Factor (NGF)-Mediated Osteogenic Differentiation of Mesenchymal Stem Cells.* Los Angeles, CA: UCLA.

[B51] Poudel S. B. Min C. Lee J. Shin Y. Kwon T. Jeon Y. (2019). Local supplementation with plant-derived recombinant human FGF2 protein enhances bone formation in critical-sized calvarial defects. *J. Bone Miner. Metab.* 37 900–912. 10.1007/s00774-019-00993-2 30843129

[B52] Qiao Q. Xu X. Song Y. Song S. Zhu W. Li F. (2018). Semaphorin 3A promotes osteogenic differentiation of BMSC from type 2 diabetes mellitus rats. *J. Mol. Histol.* 49 369–376. 10.1007/s10735-018-9776-1 29774455

[B53] Qiu J. Wang X. Zhou H. Zhang C. Wang Y. Huang J. (2020). Enhancement of periodontal tissue regeneration by conditioned media from gingiva-derived or periodontal ligament-derived mesenchymal stem cells: a comparative study in rats. *Stem Cell Res. Ther.* 11:42. 10.1186/s13287-019-1546-9 PMC6998241 32014015

[B54] Sadeghi F. Vahednia E. Naderi Meshkin H. Kerachian M. A. (2020). The effect of adrenocorticotropic hormone on alpha-2-macroglobulin in osteoblasts derived from human mesenchymal stem cells. *J. Cell. Mol. Med.* 24 4784–4790. 10.1111/jcmm.15152 32163666 PMC7176844

[B55] Seo B. Miura M. Gronthos S. Bartold P. M. Batouli S. Brahim J. (2004). Investigation of multipotent postnatal stem cells from human periodontal ligament. *Lancet* 364 149–155. 10.1016/s0140-6736(04)16627-0 15246727

[B56] Shin S. Lee J. Kwon Y. Park K. Jeong J. Choi S. (2021). Comparative proteomic analysis of the mesenchymal stem cells secretome from adipose, bone marrow, placenta and wharton’s jelly. *Int. J. Mol. Sci.* 22:845. 10.3390/ijms22020845 33467726 PMC7829982

[B57] Sirirat N. Trairak P. Thanaphum O. Prasit P. Chanin K. Sirakarnt D. (2020). Systems biology analysis of osteogenic differentiation behavior by canine mesenchymal stem cells derived from bone marrow and dental pulp. *Sci. Rep. UK* 10 20703–20703. 10.1038/s41598-020-77656-0 PMC7692528 33244029

[B58] Stein G. S. Lian J. B. Wijnen A. J. V. Stein J. L. Montecino M. Javed A. (2004). Runx2 control of organization, assembly and activity of the regulatory machinery for skeletal gene expression. *Oncogene* 23 4315–4329. 10.1038/sj.onc.1207676 15156188

[B59] Sticht C. De La Torre C. Parveen A. Gretz N. (2018). miRWalk: an online resource for prediction of microRNA binding sites. *PLoS One* 13:e0206239. 10.1371/journal.pone.0206239 30335862 PMC6193719

[B60] Sun J. She P. Liu X. Gao B. Jin D. Zhong T. P. (2021). Disruption of Abcc6 transporter in zebrafish causes ocular calcification and cardiac fibrosis. *Int. J. Mol. Sci.* 22:278. 10.3390/ijms22010278 33383974 PMC7795442

[B61] Sun Z. Yan K. Liu S. Yu X. Xu J. Liu J. (2021). Semaphorin 3A promotes the osteogenic differentiation of rat bone marrow-derived mesenchymal stem cells in inflammatory environments by suppressing the Wnt/β-catenin signaling pathway. *J. Mol. Histol.* [Epub ahead of print]. 10.1007/s10735-020-09941-1 33566267

[B62] Szklarczyk D. Franceschini A. Wyder S. Forslund K. Heller D. Huerta-Cepas J. (2015). STRING v10: protein–protein interaction networks, integrated over the tree of life. *Nucleic Acids Res.* 43 D447–D452. 25352553 10.1093/nar/gku1003 PMC4383874

[B63] Tang Y. Liu L. Wang P. Chen D. Wu Z. Tang C. (2017). Periostin promotes migration and osteogenic differentiation of human periodontal ligament mesenchymal stem cells via the Jun amino-terminal kinases (JNK) pathway under inflammatory conditions. *Cell Proliferat.* 50:e12369. 10.1111/cpr.12369 28833827 PMC6529146

[B64] Tian S. Liu Y. Dong F. Dou Y. Li W. Wang J. (2020). Knockdown of microRNA-584 promotes dental pulp stem cells proliferation by targeting TAZ. *Cell Cycle* 19 1048–1058. 10.1080/15384101.2020.1744976 32208890 PMC7217370

[B65] Ueda M. Goto T. Kuroishi K. N. Gunjigake K. K. Ikeda E. Kataoka S. (2016). Asporin in compressed periodontal ligament cells inhibits bone formation. *Arch. Oral Biol.* 62 86–92. 10.1016/j.archoralbio.2015.11.010 26655952

[B66] Urrutia D. N. Caviedes P. Mardones R. Minguell J. J. Vega-Letter A. M. Jofre C. M. (2019). Comparative study of the neural differentiation capacity of mesenchymal stromal cells from different tissue sources: an approach for their use in neural regeneration therapies. *PLoS One* 14:e0213032. 10.1371/journal.pone.0213032 30856179 PMC6437714

[B67] Ustriyana P. Schulte F. Gombedza F. Gil-Bona A. Paruchuri S. Bidlack F. B. (2021). Spatial survey of non-collagenous proteins in mineralizing and non-mineralizing vertebrate tissues ex vivo. *Bone Rep.* 2021:100754. 10.1016/j.bonr.2021.100754 33665237 PMC7900015

[B68] Wang C. Gu W. Sun B. Zhang Y. Ji Y. Xu X. (2017). CTHRC1 promotes osteogenic differentiation of periodontal ligament stem cells by regulating TAZ. *J. Mol. Histol.* 48 311–319. 10.1007/s10735-017-9729-0 28647773

[B69] Wang C. Li Y. Yu K. Jiang Z. Wang Y. Yang G. (2020). HOXA10 inhibits the osteogenic differentiation of periodontal ligament stem cells by regulating beta-catenin localization and DKK1 expression. *Connect. Tissue Res.* 62 393–401. 10.1080/03008207.2020.1756271 32299243

[B70] Wang J. Li H. Lv Z. Luo X. Deng W. Zou T. (2021). NR4A3 induces endothelial dysfunction through up-regulation of endothelial 1 expression in adipose tissue-derived stromal cells. *Life Sci.* 264:118727. 10.1016/j.lfs.2020.118727 33221345

[B71] Wang Y. Li J. Qiu Y. Hu B. Chen J. Fu T. (2018). Low-intensity pulsed ultrasound promotes periodontal ligament stem cell migration through TWIST1-mediated SDF-1 expression. *Int. J. Mol. Med.* 42 322–330. 29620151 10.3892/ijmm.2018.3592 PMC5979833

[B72] Wei F. Liu D. Feng C. Zhang F. Yang S. Hu Y. (2015). microRNA-21 mediates stretch-induced osteogenic differentiation in human periodontal ligament stem cells. *Stem Cells Dev.* 24 312–319. 10.1089/scd.2014.0191 25203845 PMC4303015

[B73] Whitfield A. J. Barrett P. H. R. Van Bockxmeer F. M. Burnett J. R. (2004). Lipid disorders and mutations in the APOB gene. *Clin. Chem.* 50 1725–1732. 10.1373/clinchem.2004.038026 15308601

[B74] Williams M. J. Sugatani T. Agapova O. A. Fang Y. Gaut J. P. Faugere M. (2018). The activin receptor is stimulated in the skeleton, vasculature, heart, and kidney during chronic kidney disease. *Kidney Int.* 93 147–158. 10.1016/j.kint.2017.06.016 28843411 PMC6628245

[B75] Xiao J. Dong H. Wu Y. Tian W. Liu L. (2009). Gene expression profiling of Cx3cl1 in bone marrow mesenchymal stem cells by osteogenic induction. *Omics* 13 337–343. 10.1089/omi.2009.0018 19422292

[B76] Xu X. He X. Wang J. Li X. Xia Y. Tan Y. (2019). Role of the P2X7 receptor in inflammation-mediated changes in the osteogenesis of periodontal ligament stem cells. *Cell Death Dis.* 10 1–17. 10.1155/2020/2016809 30622236 PMC6325129

[B77] Xu X. Y. Li X. Wang J. He X. T. Sun H. H. Chen F. M. (2019). Concise review: periodontal tissue regeneration using stem cells: strategies and translational considerations. *Stem Cell Transl. Med.* 8 392–403. 10.1002/sctm.18-0181 30585445 PMC6431686

[B78] Yamada S. Ozawa Y. Tomoeda M. Matoba R. Matsubara K. Murakami S. (2006). Regulation of PLAP-1 expression in periodontal ligament cells. *J. Dent. Res.* 85 447–451. 10.1177/154405910608500510 16632759

[B79] Yamada S. Tomoeda M. Ozawa Y. Yoneda S. Terashima Y. Ikezawa K. (2007). PLAP-1/Asporin, a novel negative regulator of periodontal ligament mineralization. *J. Biol. Chem.* 282 23070–23080. 10.1074/jbc.m611181200 17522060

[B80] Yang W. Xia Y. Qian X. Wang M. Zhang X. Li Y. (2019). Co-expression network analysis identified key genes in association with mesenchymal stem cell osteogenic differentiation. *Cell Tissue Res.* 378 513–529. 10.1007/s00441-019-03071-1 31418071

[B81] Zhang P. Zhang Y. Liu Q. Zhang Y. Ji Y. Xu X. (2020). 1,25(OH)2D3 supports the osteogenic differentiation of hPDLSCs under inflammatory conditions through inhibiting PLAP-1 expression transcriptionally. *Int. Immunopharmacol.* 78:105998. 10.1016/j.intimp.2019.105998 31837573

[B82] Zhang Y. Ding N. Zhang T. Sun Q. Han B. Yu T. (2019). A tetra-PEG hydrogel based aspirin sustained release system exerts beneficial effects on periodontal ligament stem cells mediated bone regeneration. *Front. Chem.* 7:682. 10.3389/fchem.2019.00682 31681732 PMC6811605

[B83] Zhang Y. Xing Y. Jia L. Ji Y. Zhao B. Wen Y. (2018). An in vitro comparative study of multisource derived human mesenchymal stem cells for bone tissue engineering. *Stem Cells Dev.* 27 1634–1645. 10.1089/scd.2018.0119 30234437

[B84] Zhang Y. Zheng J. Zhou Z. Zhou H. Wang Y. Gong Z. (2015). Fractalkine promotes chemotaxis of bone marrow-derived mesenchymal stem cells towards ischemic brain lesions through Jak2 signaling and cytoskeletal reorganization. *FEBS J.* 282 891–903. 10.1111/febs.13187 25559502

