# Supplementary figures and images for: Bioinformatics Analysis Identified miR-584-5p and Key miRNA-mRNA Networks Involved in the Osteogenic Differentiation of Human Periodontal Ligament Stem Cells
Source: Front Genet. 2021 Sep 27;12:750827. doi: 10.3389/fgene.2021.750827 (PMC8503254; doi:10.3389/fgene.2021.750827)

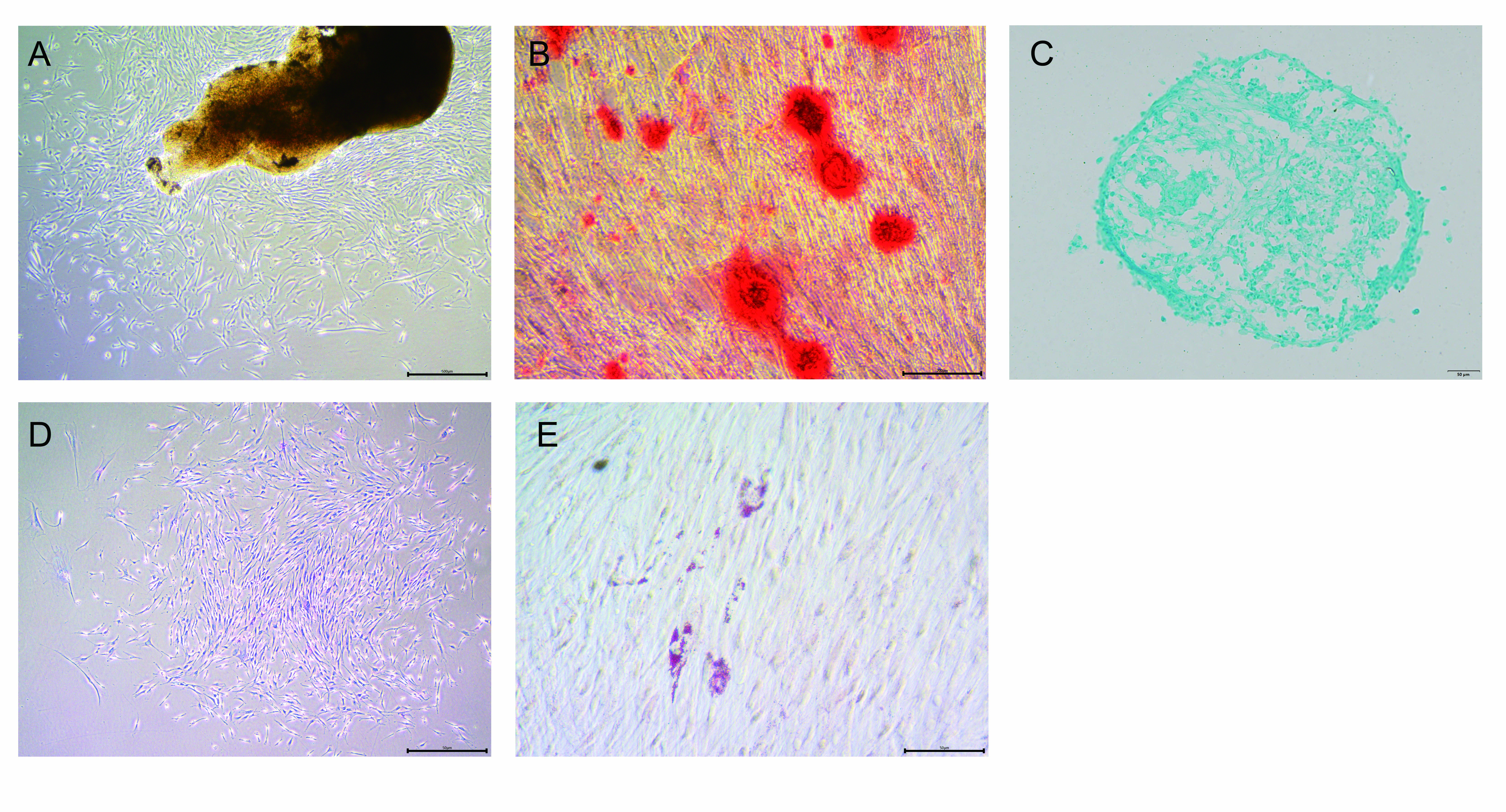

Supplement: Supplementary Figure 1 — Isolation and characterization of human PDLSCs. PDLSCs at P0 showed a typical fibroblast-like spindle appearance (A). Mineralized nodules were stained by ARS at 14d (B), cartilage tissue was stained by Alcian Blue at 21d (C), Clone formation (D), and lipid droplets were stained by Oil Red O staining at 14d (E). [file Data_Sheet_1.zip › Figure S1.jpg]
